# Lung tissue shows divergent gene expression between chronic obstructive pulmonary disease and idiopathic pulmonary fibrosis

**DOI:** 10.1186/s12931-022-02013-w

**Published:** 2022-04-21

**Authors:** Auyon J. Ghosh, Brian D. Hobbs, Jeong H. Yun, Aabida Saferali, Matthew Moll, Zhonghui Xu, Robert P. Chase, Jarrett Morrow, John Ziniti, Frank Sciurba, Lucas Barwick, Andrew H. Limper, Kevin Flaherty, Gerard Criner, Kevin K. Brown, Robert Wise, Fernando J. Martinez, Daniel McGoldrick, Michael H. Cho, Dawn L. DeMeo, Edwin K. Silverman, Peter J. Castaldi, NHLBI Trans-Omics for Precision Medicine (TOPMed) Consortium, James D. Crapo, Edwin K. Silverman, Barry J. Make, Elizabeth A. Regan, Terri Beaty, Ferdouse Begum, Peter J. Castaldi, Michael Cho, Dawn L. DeMeo, Adel R. Boueiz, Marilyn G. Foreman, Eitan Halper-Stromberg, Lystra P. Hayden, Craig P. Hersh, Jacqueline Hetmanski, Brian D. Hobbs, John E. Hokanson, Nan Laird, Christoph Lange, Sharon M. Lutz, Merry-Lynn McDonald, Margaret M. Parker, Dmitry Prokopenko, Dandi Qiao, Phuwanat Sakornsakolpat, Emily S. Wan, Sungho Won, Juan Pablo Centeno, Jean-Paul Charbonnier, Harvey O. Coxson, Craig J. Galban, MeiLan K. Han, Eric A. Hoffman, Stephen Humphries, Francine L. Jacobson, Philip F. Judy, Ella A. Kazerooni, Alex Kluiber, David A. Lynch, Pietro Nardelli, John D. Newell Jr, Aleena Notary, Andrea Oh, James C. Ross, Raul San Jose Estepar, Joyce Schroeder, Jered Sieren, Berend C. Stoel, Juerg Tschirren, Edwin Van Beek, Bram van Ginneken, Eva van Rikxoort, Gonzalo Vegas Sanchez-Ferrero, Lucas Veitel, George R. Washko, Carla G. Wilson, Robert Jensen, Douglas Everett, Jim Crooks, Katherine Pratte, Matt Strand, Gregory Kinney, Kendra A. Young, Surya P. Bhatt, Jessica Bon, Alejandro A. Diaz, Susan Murray, Xavier Soler, Russell P. Bowler, Katerina Kechris, Farnoush Banaei-Kashani, Jeffrey L. Curtis, Perry G. Pernicano, Nicola Hanania, Mustafa Atik, Aladin Boriek, Kalpatha Guntupalli, Elizabeth Guy, Amit Parulekar, R. Graham Barr, John Austin, Belinda D’Souza, Byron Thomashow, Neil MacIntyre, H. Page McAdams, Lacey Washington, Eric Flenaugh, Silanth Terpenning, Charlene McEvoy, Joseph Tashjian, Robert Wise, Robert Brown, Nadia N. Hansel, Karen Horton, Allison Lambert, Nirupama Putcha, Richard Casaburi, Alessandra Adami, Matthew Budoff, Hans Fischer, Janos Porszasz, Harry Rossiter, William Stringer, Amir Sharafkhaneh, Charlie Lan, Christine Wendt, Brian Bell, Ken M. Kunisaki, Richard Rosiello, David Pace, Gerard Criner, David Ciccolella, Francis Cordova, Chandra Dass, Gilbert D’Alonzo, Parag Desai, Michael Jacobs, Steven Kelsen, Victor Kim, A. James Mamary, Nathaniel Marchetti, Aditi Satti, Kartik Shenoy, Robert M. Steiner, Alex Swift, Irene Swift, Maria Elena Vega-Sanchez, Mark Dransfield, William Bailey, Anand Iyer, Hrudaya Nath, J. Michael Wells, Douglas Conrad, Andrew Yen, Alejandro P. Comellas, Karin F. Hoth, Brad Thompson, Wassim Labaki, Dharshan Vummidi, Joanne Billings, Abbie Begnaud, Tadashi Allen, Frank Sciurba, Divay Chandra, Carl Fuhrman, Joel Weissfeld, Antonio Anzueto, Sandra Adams, Diego Maselli-Caceres, Mario E. Ruiz, Harjinder Sing, Craig P. Hersh

**Affiliations:** 1 Channing Division of Network Medicine, Department of Medicine, Brigham and Women’s Hospital, 181 Longwood Avenue, Boston, MA 02115 USA; 2 Division of Pulmonary and Critical Care Medicine, Department of Medicine, Brigham and Women’s Hospital, Boston, MA USA; 3 Harvard Medical School, Boston, MA USA; 4 Division of Pulmonary, Allergy and Critical Care Medicine, Department of Medicine, University of Pittsburgh, Pittsburgh, PA USA; 5 The Emmes Company, Rockville, MD USA; 6 Division of Pulmonary and Critical Care Medicine, Department of Internal Medicine, Mayo Clinic, Rochester, MN USA; 7 Division of Pulmonary and Critical Care Medicine, Department of Internal Medicine, University of Michigan Healthy System, Ann Arbor, MI USA; 8 Department of Thoracic Medicine and Surgery, Temple University, Philadelphia, PA USA; 9 Department of Medicine, National Jewish Health, Denver, CO USA; 10 Division of Pulmonary and Critical Care Medicine, Johns Hopkins School of Medicine, Baltimore, MD USA; 11 Division of Pulmonary and Critical Care Medicine, Department of Medicine, Weill Cornell Medicine, New York, NY USA; 12 Northwest Genomics Center, University of Washington, Seattle, WA USA

**Keywords:** COPD, IPF, RNA sequencing

## Abstract

**Background:**

Chronic obstructive pulmonary disease (COPD) and idiopathic pulmonary fibrosis (IPF) are characterized by shared exposures and clinical features, but distinct genetic and pathologic features exist. These features have not been well-studied using large-scale gene expression datasets. We hypothesized that there are divergent gene, pathway, and cellular signatures between COPD and IPF.

**Methods:**

We performed RNA-sequencing on lung tissues from individuals with IPF (n = 231) and COPD (n = 377) compared to control (n = 267), defined as individuals with normal spirometry. We grouped the overlapping differential expression gene sets based on direction of expression and examined the resultant sets for genes of interest, pathway enrichment, and cell composition. Using gene set variation analysis, we validated the overlap group gene sets in independent COPD and IPF data sets.

**Results:**

We found 5010 genes differentially expressed between COPD and control, and 11,454 genes differentially expressed between IPF and control (1% false discovery rate). 3846 genes overlapped between IPF and COPD. Several pathways were enriched for genes upregulated in COPD and downregulated in IPF; however, no pathways were enriched for genes downregulated in COPD and upregulated in IPF. There were many myeloid cell genes with increased expression in COPD but decreased in IPF. We found that the genes upregulated in COPD but downregulated in IPF were associated with lower lung function in the independent validation cohorts.

**Conclusions:**

We identified a divergent gene expression signature between COPD and IPF, with increased expression in COPD and decreased in IPF. This signature is associated with worse lung function in both COPD and IPF.

**Supplementary Information:**

The online version contains supplementary material available at 10.1186/s12931-022-02013-w.

## Background

Chronic obstructive pulmonary disease (COPD) is a chronic lung disease characterized by airflow limitation, airway inflammation, and lung parenchymal destruction. In addition to well-known COPD risk factors including age and cigarette smoking, more than 80 genetic loci have been associated with COPD susceptibility [1, 2]. Idiopathic pulmonary fibrosis (IPF) is another chronic lung disease with age and smoking as risk factors but is characterized by lung parenchymal scarring on pathology and imaging and restriction on lung function testing. While fewer genetic loci have been associated with IPF, the estimated heritability is greater for IPF compared to COPD, largely due to variants in the *MUC5B* gene.

Given the shared non-genetic risk factors, there has been significant recent interest in identifying shared genetic factors and biological mechanisms that underlie the development and progression of both diseases. Genome-wide association studies (GWAS) have identified five genetic loci that overlap between IPF and COPD [3, 4], albeit in opposite direction of association. Previous studies using gene expression from lung tissue have demonstrated enrichment of the p53/hypoxia pathway among the overlapped differentially expressed genes and alternative splice variants in COPD and IPF [5]. In addition, several studies have implicated accelerated cellular senescence in the development of both diseases [6, 7].

While convergent genes and pathways have been demonstrated, fewer studies have investigated the divergent genes, pathways, and cell populations represented in the contrast of the disease states. Epithelial [8] vs. mesenchymal [9] precursor cell dysfunction has been linked to the divergence of COPD and IPF [10]. In addition, the Wnt and Notch signaling pathways appear to be overactivated in IPF but aberrantly inhibited in COPD [11, 12]. However, the larger context of the genes and pathways involved and their relative effects in each disease is less clear. We therefore sought to identify genes and pathways involved in the divergence of IPF and COPD using gene expression in resected human lung tissue from well-characterized subjects. We additionally hypothesized that these divergent gene sets are associated with worse clinical outcomes in both COPD and IPF.

## Methods

### Study participants

Lung tissue samples were obtained through the Lung Tissue Research Consortium (LTRC). Further details regarding subject recruitment have been previously published [13]. Institutional review boards approved the study at all participating institutions and all patients provided written informed consent per LTRC protocol. We made the diagnosis of IPF based on a consensus clinical diagnosis of IPF according to American Thoracic Society/European Respiratory Society guidelines for diagnosis of IPF or a pathologic diagnosis of UIP or honeycomb lung [14]. We defined COPD as forced expiratory volume in one second (FEV_1_) to forced vital capacity (FVC) ratio < 0.70 and FEV_1_% predicted  < 80% (Global Initiative for Chronic Obstructive Lung Disease [GOLD] stage 2–4) [15] and either pathologic emphysema or no alternative pathologic diagnosis. We defined control subjects as FEV_1_/FVC ratio ≥ 0.70 and either no alternative pathologic diagnosis or pathologic emphysema. We excluded subjects missing demographic information, including current smoking status, and subjects whose corresponding tissue did not have pathology information for the lobe of origin.

Validation of findings from LTRC was performed using blood RNA-seq data from the COPDGene study [16] and blood gene expression microarrays from a study of IPF subjects initially recruited to investigate host-microbial interactions in IPF [17].

### Differential expression

The methods of RNA data processing are available in Additional file 1. We performed differential expression using the limma and voom packages. Models were adjusted for age, race, sex, current smoking status, smoking pack-years, and library preparation batch. Surrogate variables were used to estimate other latent effects and included in the models as covariates. Multiple testing was controlled with a false discovery rate (FDR) < 1%.

Genes that were differentially expressed in both COPD and IPF were grouped into four groups based on direction of log_2_ fold change, where Group 1 was defined by genes with increased expression in IPF and COPD and Group 4 was defined by genes with decreased expression in IPF and COPD (convergent gene sets), and Group 2 was defined by genes with increased expression in IPF but decreased expression in COPD and Group 3 was defined by genes with decreased expression in IPF but increased expression in COPD (divergent gene sets). The methods for gene set enrichment, pathway analysis, cell category composition, and cell deconvolution are available in Additional file 1.

### Gene set variation analysis

To investigate the association of our overlap expression gene sets with outcomes in LTRC including FEV_1_ and DLCO, we employed gene set variation analysis (GSVA) [18].

GSVA can be used to reduce noise and improve interpretability via dimension reduction of the overlap gene expression sets [19]. GSVA produces an enrichment score, similar to those in other gene set enrichment techniques, but allows for analysis of novel gene sets not necessarily defined by pathways or other externally pre-determined gene sets. The scores can then be tested for association with various phenotypic data using traditional statistical methods. We tested the association of overlap gene expression set GSVA score with clinical outcomes in all subjects from LTRC. We validated these findings using blood RNA sequencing data and clinical and chest CT imaging outcomes from an independent COPD case–control study [20] and blood RNA sequencing data and clinical outcomes from a publicly available IPF case–control study [17] (GEO accession GSE93606) and validated the association of the overlap gene sets with disease status in each replication cohort.

## Results

### Subject characteristics

We obtained lung samples from 1503 subjects from the LTRC. 1399 subjects had complete demographic and pathology information. Of these, 231 subjects met case criteria for IPF, 377 subjects met case criteria for COPD, and 267 subjects met control criteria. Demographic and clinical characteristics for the subjects included in the present study are shown in Table 1.

**Table 1:** LTRC subject characteristics

Characteristic	IPF	COPD	Control	*p* value IPF vs. control	*p* value COPD vs. control
n	231	377	267		
Age, years	63.39 (7.90)	63.78 (9.05)	61.90 (12.65)	0.123	0.029
Male sex	157 (68.0)	201 (53.3)	105 (39.3)	< 0.001	0.001
Race				0.180	0.222
White	205 (88.7)	344 (91.2)	246 (92.1)		
Asian	3 (1.3)	0 (0)	0 (0)		
Black	10 (4.3)	26 (6.9)	11 (4.1)		
Hispanic	7 (3.0)	5 (1.3)	8 (3.0)		
Other race	6 (2.6)	2 (0.5)	2 (0.7)		
BMI, kg/m^2^	29.72 (5.44)	26.03 (5.11)	28.75 (5.88)	0.060	< 0.001
Current smoking	3 (1.3)	25 (6.6)	10 (3.7)	0.154	0.157
Ever smoking	152 (65.8)	365 (96.8)	173 (64.8)	0.888	< 0.001
Smoking pack-years	18.06 (23.33)	47.76 (30.48)	19.11 (26.75)	0.642	< 0.001
Pre-bronchodilator FEV_1_, % predicted	66.17 (19.28)	40.18 (19.74)	96.62 (12.83)	< 0.001	< 0.001
Pre-bronchodilator FVC, % predicted	61.11 (18.06)	67.94 (18.11)	96.67 (13.10)	< 0.001	< 0.001
Pre-bronchodilator FEV_1_/FVC	0.83 (0.08)	0.43 (0.15)	0.77 (0.06)	< 0.001	< 0.001
DLCO, % predicted	38.95 (19.80)	40.98 (18.22)	73.78 (15.52)	< 0.001	< 0.001

Data presented as mean (SD) or no. (%)

*LTRC* Lung Tissue Research Consortium, *IPF* idiopathic pulmonary fibrosis, *COPD* chronic obstructive pulmonary disease, *BMI* body mass index, *FEV*_*1*_ forced expiratory volume in 1 s, *FVC* forced vital capacity, *DLCO* diffusion capacity for carbon monoxide

Compared to controls, IPF subjects were more likely to be male but otherwise similar demographically. Compared to controls, COPD subjects were older, more likely to be male, had lower BMI, and significantly higher lifetime smoking intensity in pack-years.

### Differential gene expression

**Fig. 1:**
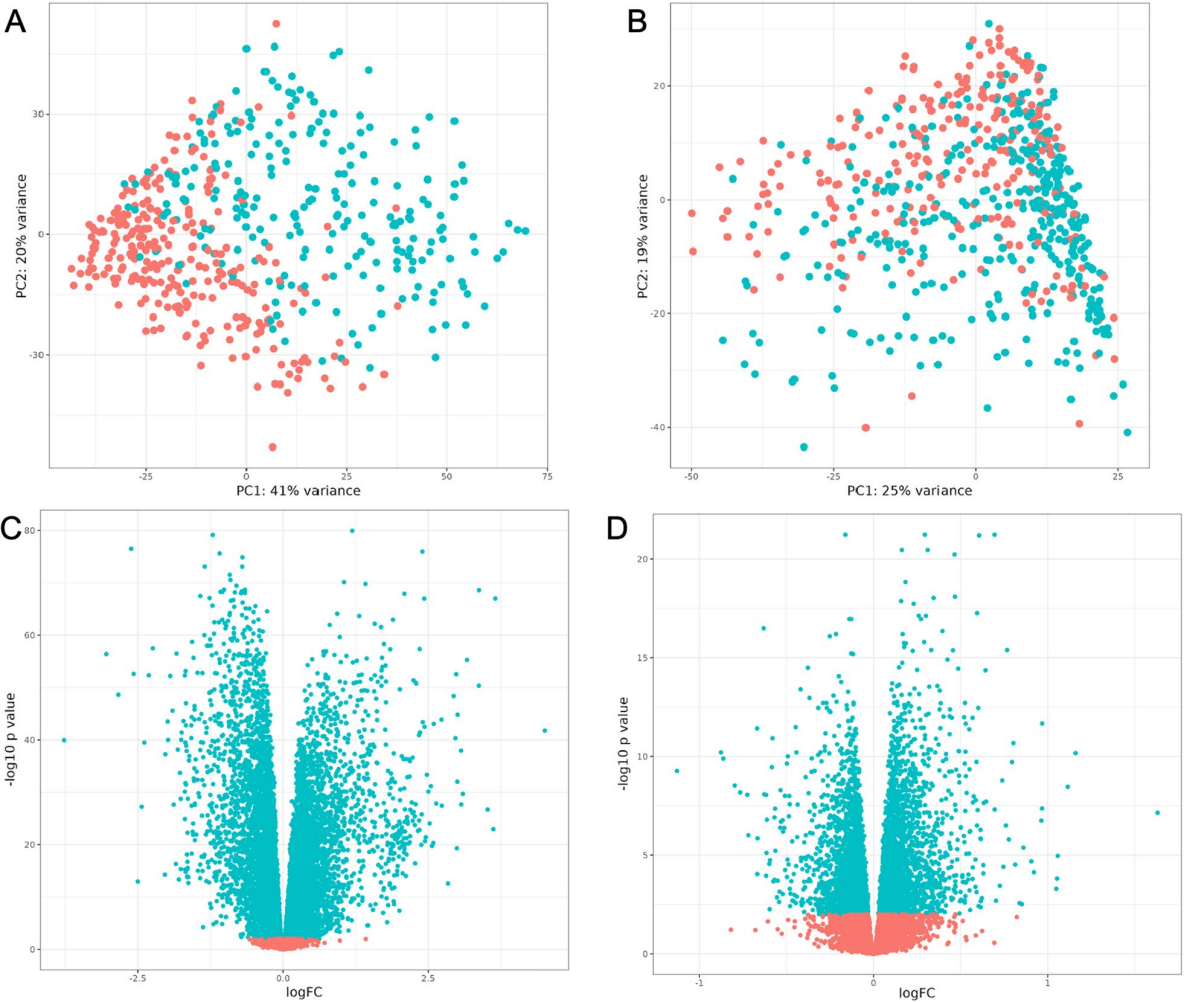
Principal component analysis (PCA) plots for lung tissue RNA sequencing data and volcano plots of differential expression results. Principal component analysis (PCA) plots for lung tissue RNA sequencing data and volcano plots of differential expression results. **A** PCA plot for control samples (orange) vs. IPF samples (blue). **B** PCA plot for control samples (orange) vs. COPD samples (blue). **C** Volcano plot of IPF vs. control differential expression results. FDR < 0.01 results shown in blue and results that did not meet FDR threshold are shown in orange. **D** Volcano plot of COPD vs. control differential expression results

**Fig. 2:**
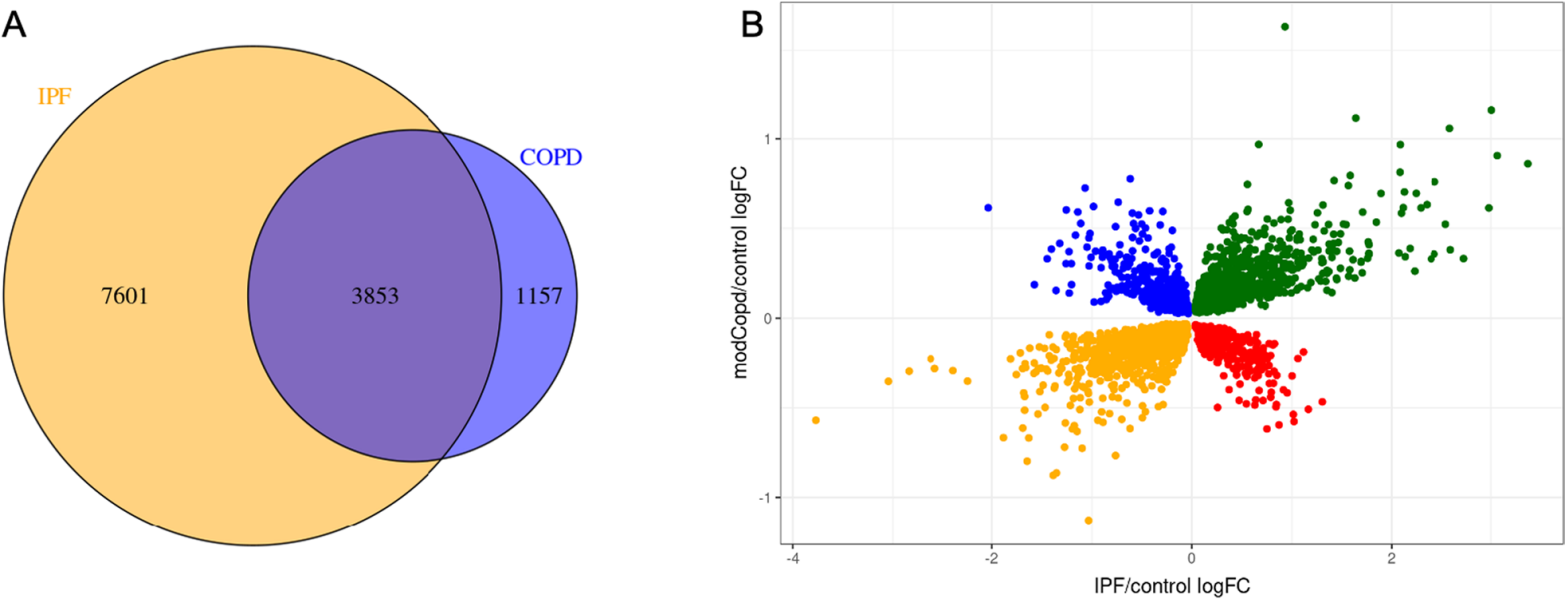
Overlap of IPF and COPD vs. control differentially expressed genes. **A** Venn diagram of differentially expressed genes between IPF vs. control and COPD vs. control. **B** Scatterplot of overlapping differentially expressed genes with log_2_ fold change of IPF vs. control on *x* axis and log_2_ fold change of COPD vs. control on *y* axis. Genes with increased expression in IPF and COPD (Group 1) are in green; genes with increased expression in IPF but decreased expression in COPD (Group 2) are in red; genes with decreased expression in IPF but increased expression in COPD (Group 3) are in blue; genes with decreased expression in IPF and COPD (Group 4) are in orange

There were 58,870 RNAs identified among all subjects. After filtering for low expressed genes, we assessed 15,893 genes for differential expression between IPF and controls and 15,578 genes for differential expression between COPD and controls (Fig. 1). We found 11,454 and 5010 genes differentially expressed for IPF and COPD, respectively, after accounting for multiple testing using 1% FDR (Additional file 2: Tables S1 and S2). There were 3853 genes that overlapped between IPF and COPD (Fig. 2A, Additional file 2: Table S3). Of these, 3846 genes had corresponding HGNC symbols and were used in downstream analyses. There were 1507 genes in Group 1 (upregulated in both COPD and IPF), 397 genes in Group 2 (downregulated in COPD, upregulated in IPF), 530 genes in Group 3 (upregulated in COPD, downregulated in IPF), and 1412 genes in Group 4 (downregulated in in both) (Fig. 2B).

### Gene set enrichment and pathway analysis

We examined the differential expression of the nearest genes and all genes within 200 kb of the 82 top single nucleotide polymorphisms identified in a large, collaborative GWAS of COPD [1]. 18 of the nearest genes were differentially expressed (1% FDR) (Table 2), while 64 out of 490 genes within 400 kb (± 200 kb) were differentially expressed (Additional file 2: Table S4). We also examined the 4 genes, which were identified through chromatin interaction, methylation, open chromatin regions, and deleterious coding variants, affected by COPD expression quantitative trait loci (eQTLs). Of these 4 genes, 2 genes (*ADAMTSL3* and *RIN3*) were present in the overlapping differentially expressed genes between IPF and COPD.

**Table 2:** COPD GWAS [1] genes in the overlapping differentially expressed gene groups

Gene	COPD vs. control log_2_FC	Unadjusted *p* value	Adjusted *p* value	Overlap group
TGFB2	− 0.32	1.72E−15	3.51E−13	4
ZKSCAN1	− 0.14	8.44E−12	4.59E−10	2
PABPC4	0.14	1.99E−10	6.96E−09	1
MFAP2	0.27	1.87E−07	2.44E−06	1
ADAMTSL3*	− 0.18	4.48E−07	5.21E−06	4
AGER	− 0.23	5.32E−07	6.03E−06	4
PID1	0.16	5.00E−07	5.72–06	3
RPL23	0.11	9.15E−07	9.56–06	1
MFHAS1	0.11	1.22E−06	1.22E−05	3
BTC	− 0.17	2.27E−05	1.47E−04	2
CHRM3	− 0.28	4.39E−05	2.60E−04	4
SFTPD	0.17	1.10E−04	5.80E−04	3
MED13L	− 0.06	1.04E−04	5.47E−04	4
NR4A2	0.45	3.20E−04	1.42E−03	3
ZBTB38	− 0.06	3.40E−04	1.48E−03	4
HHIP	0.19	1.73E−03	5.92E−03	3
RIN3*	0.07	2.09E−03	6.93E−03	3
CITED2	0.16	2.21E−03	7.28E−03	3

The gene nearest to each lead GWAS single nucleotide polymorphism is included

COPD vs. control log_2_FC, unadjusted p values, and Benjamini–Hochberg adjusted p value shown. Genes identified as eQTLs denoted with *. Group 1: genes with increased expression in IPF and COPD; Group 2: genes with increased expression in IPF but decreased expression in COPD; Group 3: genes with decreased expression in IPF but increased expression in COPD; Group 4: genes with decreased expression in IPF and COPD

*COPD* chronic obstructive pulmonary disease, *GWAS* genome-wide association study, *log*_*2*_*FC* log_2_ fold change, *eQTL* expression quantitative trait locus

Several genes had decreased expression in COPD compared to control, including *TGFB2*, *ADAMTSL3*, and *AGER*. We examined the differential expression of the nearest genes and genes within 200 kb of the 13 genetic loci identified in a GWAS of IPF. Three of the nearest genes were differentially expressed in IPF vs. controls (1% FDR), including *AKAP13*, *DEPTOR*, and *DPP9* (Table 3), while 19 of the 125 genes within 200 kb were differentially expressed (Additional file 2: Table S5).

**Table 3:** IPF GWAS [4] genes in the overlapping differentially expressed gene groups

Gene	IPF vs. control log_2_FC	Unadjusted *p* value	Adjusted *p* value	Overlap group
AKAP13	−0.25	3.48E−21	1.88E−20	3
DEPTOR	0.33	2.13E−06	4.07E−06	1
DPP9	0.08	1.43E−05	2.56E−05	1

The gene nearest to each lead GWAS single nucleotide polymorphism is included

IPF vs. control log_2_FC and Benjamini–Hochberg adjusted p value shown. Group 1: genes with increased expression in IPF and COPD; Group 2: genes with increased expression in IPF but decreased expression in COPD; Group 3: genes with decreased expression in IPF but increased expression in COPD; Group 4: genes with decreased expression in IPF and COPD

*IPF* idiopathic pulmonary fibrosis, *GWAS* genome-wide association study, *log*_*2*_*FC* log_2_ fold change

**Fig. 3:**
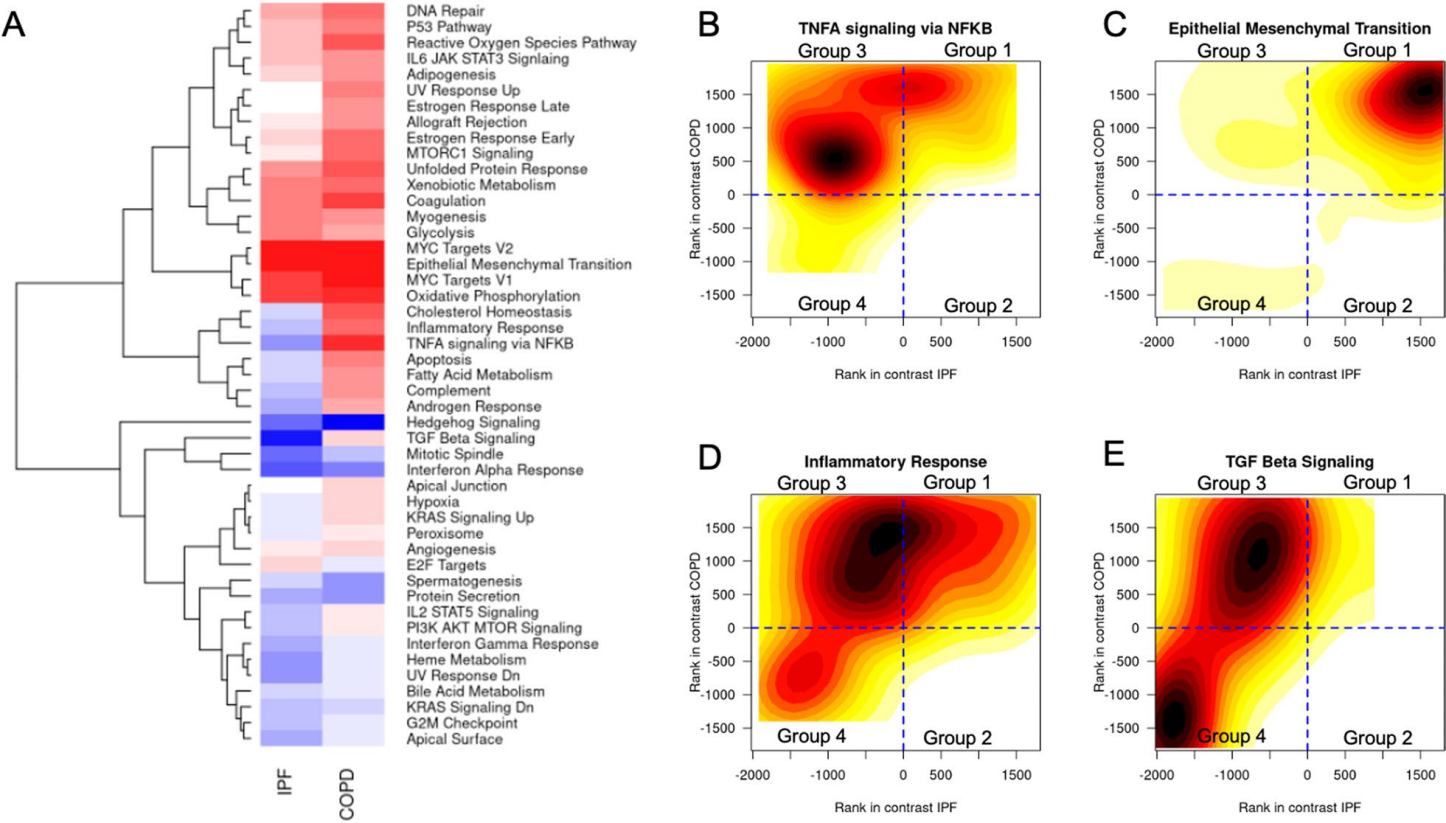
Hallmark pathway enrichment. **A** Heatmap of enrichment scores for differentially expressed genes in IPF and COPD vs. control with hierarchical clustering. Cells in red denote positive enrichment score whereas cells in blue denote negative enrichment score. **B** Density plot of genes ranks in IPF on *x* axis and gene ranks in COPD on *y* axis in TNF-alpha signaling via NFKB pathway. Color gradient represents the number of genes at the given rank coordinates, where darker shades of red denote the location with the highest number of genes and lighter shades of yellow denote the location with the lowest number genes. **C** Density plot of genes ranks in IPF on *x* axis and gene ranks in COPD on *y* axis in epithelial mesenchymal transition pathway. **D** Density plot of gene ranks in IPF on *x* axis and gene ranks in COPD on *y* axis in inflammatory response pathway. **E** Density plot of gene ranks in IPF on *x* axis and gene ranks in COPD on *y* axis in TGF Beta signaling pathway

Of the 50 MSigDB Hallmark pathways, 31 pathways demonstrated significant enrichment in the four COPD and IPF overlap gene groups (Additional file 2: Table S6). In the convergent gene sets, there were 16 pathways significantly enriched in Group 1 (upregulated in both COPD and IPF) and 5 pathways enriched in Group 4 (downregulated in both COPD and IPF). In the divergent gene sets, there were 0 pathways enriched in Group 2 (downregulated in COPD, upregulated in IPF) but there were 10 pathways significantly enriched in Group 3 (upregulated in COPD, downregulated in IPF). A heatmap of the enrichment scores and hierarchical clustering of the pathways is shown in Fig. 3A. The top enriched pathways, ranked by joint enrichment MANOVA p value, include tumor necrosis factor alpha (TNFA) signaling, epithelial–mesenchymal transition, inflammatory response, and transforming growth factor beta (TGFB) signaling. While the majority of pathways showed single visual clusters of genes, TGFB signaling and TNFA signaling appeared to have two separate clusters (Fig. 3B–E). TGFB signaling, TNFA signaling, and inflammatory response were in Group 3, while epithelial–mesenchymal transition was in Group 1.

### Cell category composition of overlap groups and cell type deconvolution

**Fig. 4:**
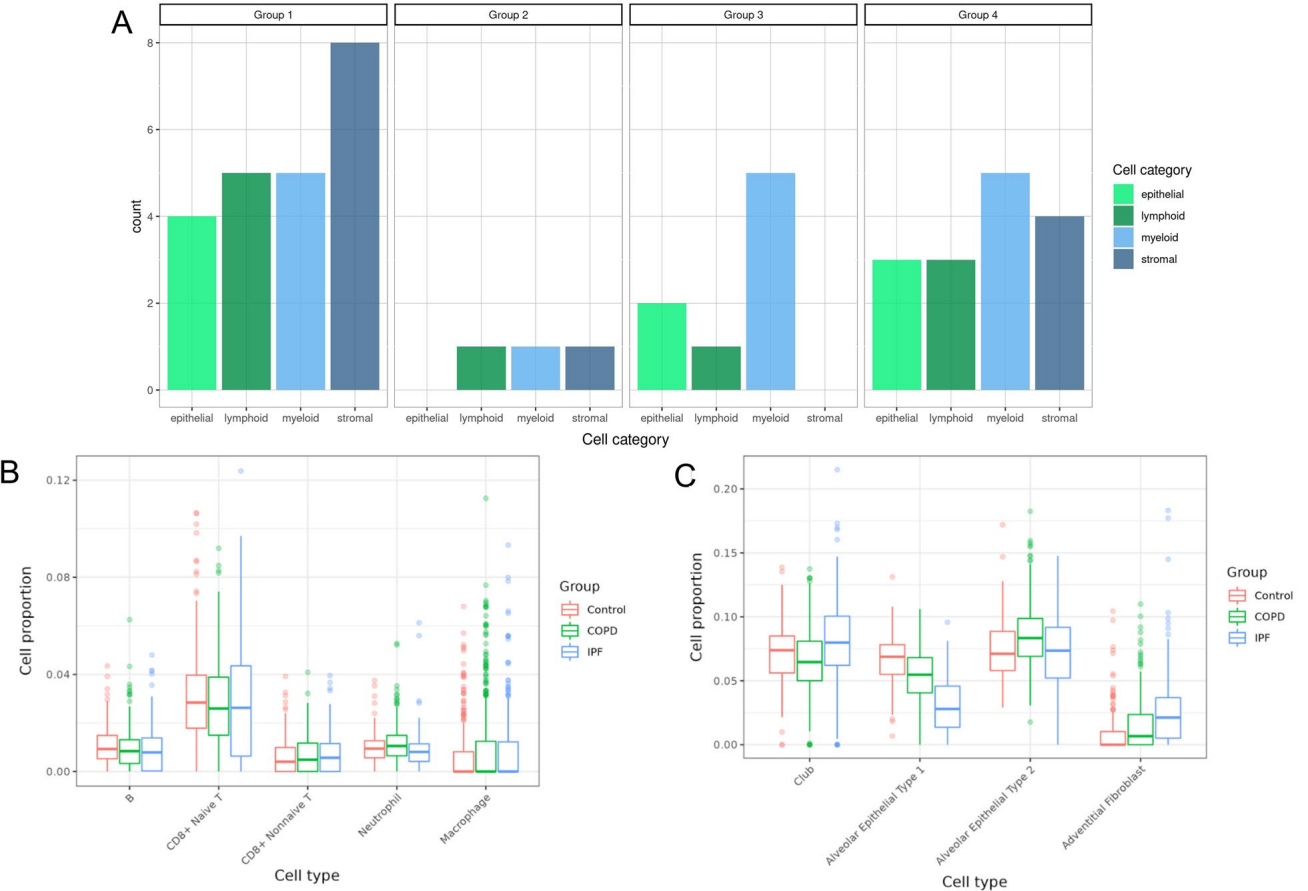
Cell category composition of differentially expressed genes and cell deconvolution in IPF and COPD. **A** Histogram of differentially expressed genes in LTRC in each overlap group by single cell RNA sequencing (scRNASeq) defined cell category [26]. Cell category genes were defined by top 5 scRNASeq genes differentially expressed by each cell type within each category. Group 1: genes with increased expression in IPF and COPD; Group 2: genes with increased expression in IPF but decreased expression in COPD; Group 3: genes with decreased expression in IPF but increased expression in COPD; Group 4: genes with decreased expression in IPF and COPD. **B** Selected COPD-associated deconvoluted cell type proportion distributions across COPD, IPF, and control samples. **C** Selected IPF-associated deconvoluted cell type proportion distributions across COPD, IPF, and control samples

Using the top five genes ranked by FDR adjusted p-value from a previously reported single cell RNA-Seq study in IPF and COPD [21] for each of the 38 cell types in four cell categories, we examined the distribution of the 190 genes in the four overlap groups. There was an overrepresentation of stromal cell genes in Group 1 and a relatively uniform distribution of cell categories in Group 4. In the divergent gene sets, there was minimal representation of lymphoid, myeloid, and stromal cell genes in Group 2 and an overrepresentation of myeloid genes in Group 3 (Fig. 4A).

We performed cell type deconvolution using Bisque, a robust and efficient method that employs non-negative least-squares regression, and a publicly available lung single-cell RNA sequencing data [19, 22]. We focused on cell types previously associated with COPD and IPF [23, 24]. We found that there was a higher proportion of adventitial fibroblasts (p = 0.035) in IPF samples compared to COPD and control samples, but there was no difference between the other selected cell types (Fig. 4B, C).

### Gene set variation analysis in LTRC

**Fig. 5:**
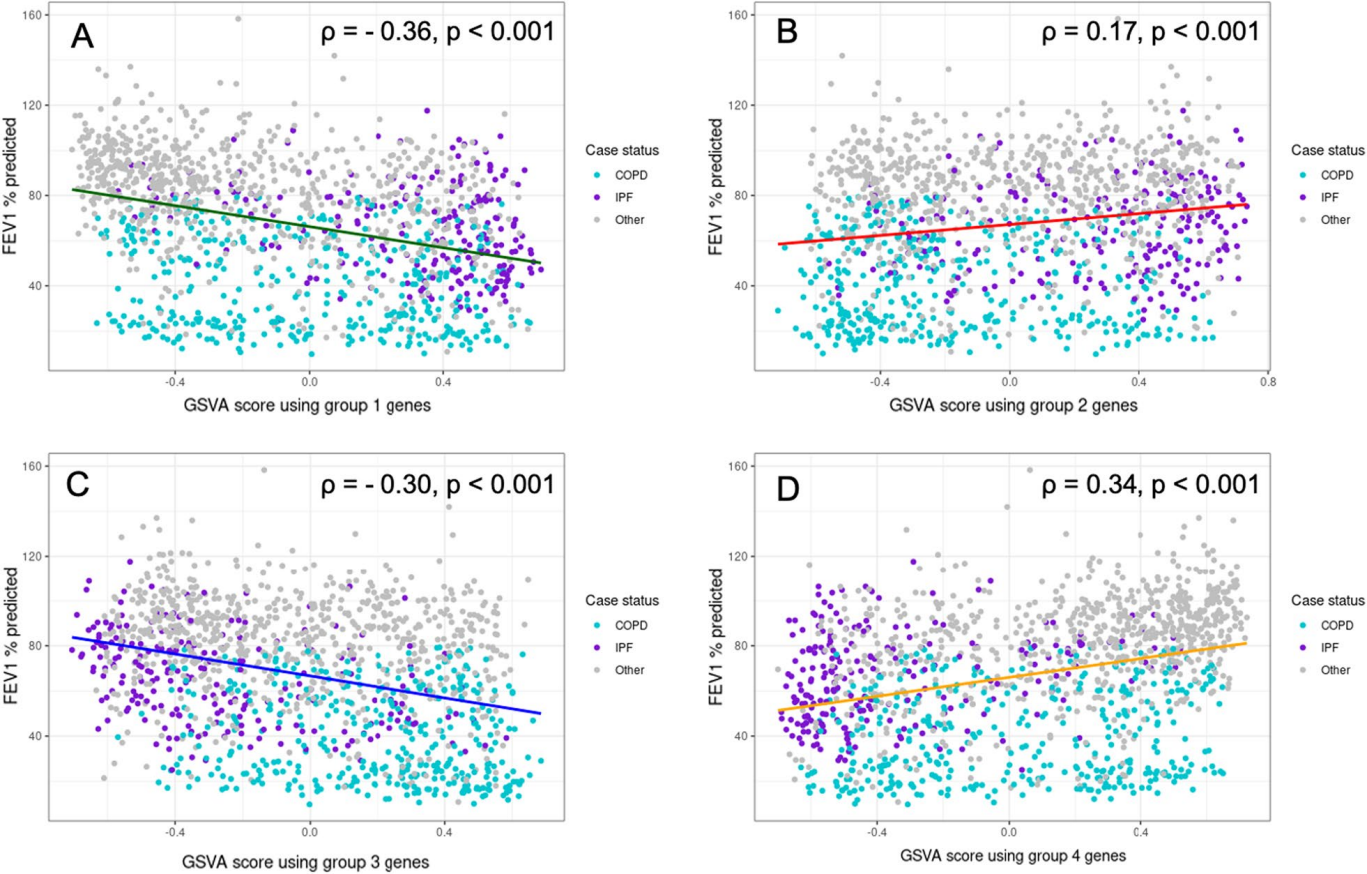
Association of GSVA scores from overlap groups with FEV_1_% predicted in LTRC. Scatterplots with trend lines of association of GSVA scores from overlap groups with FEV_1_% predicted in LTRC subjects. Turquoise represents COPD cases, purple represents IPF cases, and gray represents all other diagnoses. Trend lines are colored to represent overlap group. Spearman correlation coefficient and p value are shown for each association. Group 1: genes with increased expression in IPF and COPD; Group 2: genes with increased expression in IPF but decreased expression in COPD; Group 3: genes with decreased expression in IPF but increased expression in COPD; Group 4: genes with decreased expression in IPF and COPD. **A** Association of GSVA score from Group 1 genes with FEV_1_% predicted. **B** Association of GSVA score from Group 2 genes with FEV_1_% predicted. **C** Association of GSVA score from Group 3 with FEV_1_% predicted. **D** Association of GSVA score from Group 4 with FEV_1_% predicted. *GSVA* gene set variation analysis, *LTRC* Lung Tissue Research Consortium, *COPD* chronic obstructive pulmonary disease, *IPF* idiopathic pulmonary fibrosis, *FEV*_*1*_ forced expiratory volume over 1 s

**Fig. 6:**
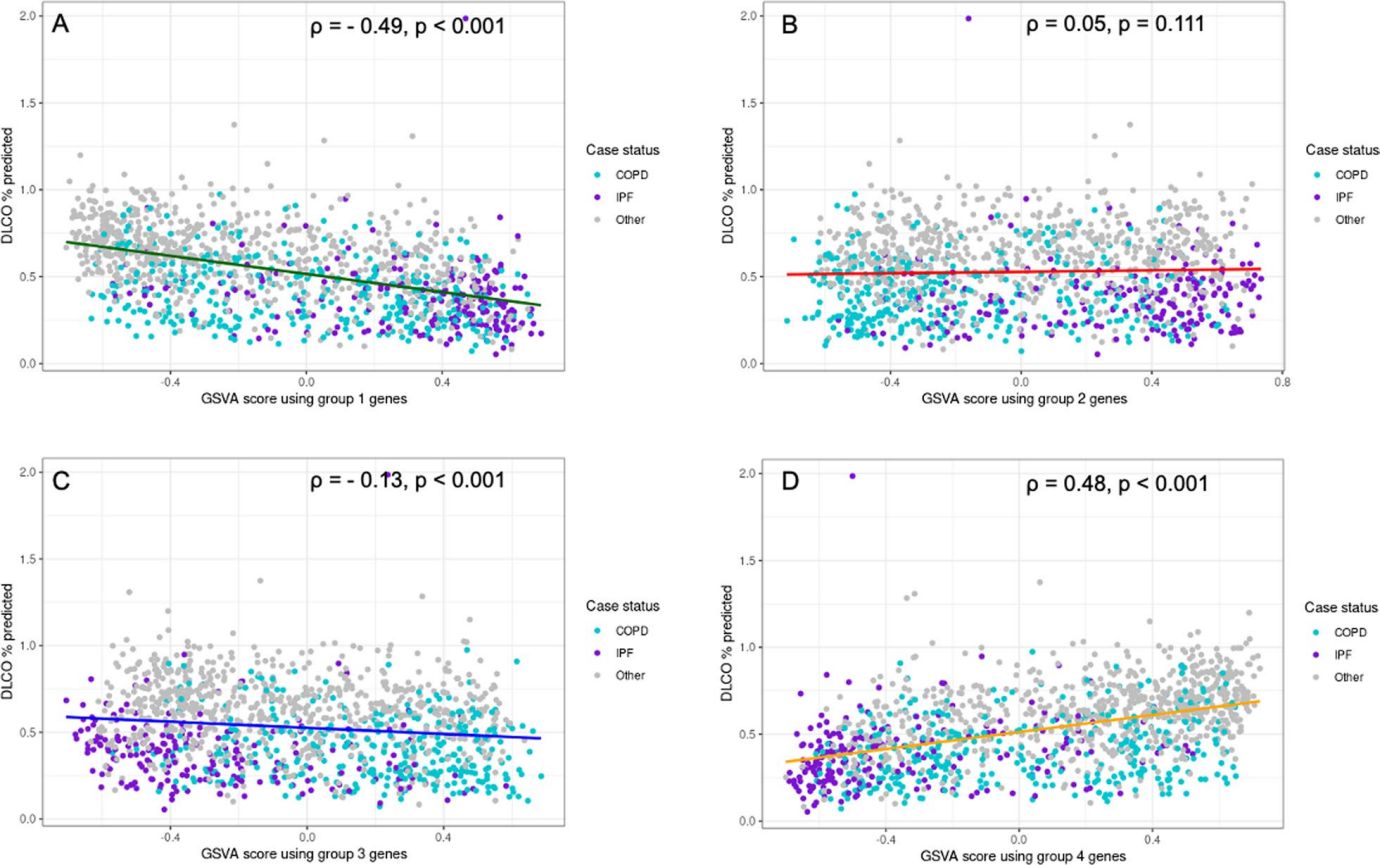
Association of GSVA scores from overlap groups with DLCO % predicted in LTRC. Scatterplots with trend lines of association of GSVA scores from overlap groups with DLCO % predicted in LTRC subjects. Turquoise represents COPD cases, purple represents IPF cases, and gray represents all other diagnoses. Trend lines are colored to represent overlap group. Spearman correlation coefficient and p value are shown for each association. Group 1: genes with increased expression in IPF and COPD; Group 2: genes with increased expression in IPF but decreased expression in COPD; Group 3: genes with decreased expression in IPF but increased expression in COPD; Group 4: genes with decreased expression in IPF and COPD. **A** Association of GSVA score from Group 1 genes with DLCO % predicted. **B** Association of GSVA score from Group 2 genes with DLCO % predicted. **C** Association of GSVA score from Group 3 with DLCO % predicted. **D** Association of GSVA score from Group 4 with DLCO % predicted. *GSVA* gene set variation analysis, *LTRC* Lung Tissue Research Consortium, *COPD* chronic obstructive pulmonary disease, *IPF* idiopathic pulmonary fibrosis, *DLCO* diffusion capacity of carbon monoxide

We used GSVA to compute scores for the differentially expressed genes from Groups 1–4. The scores were applied in all LTRC subjects, including COPD subjects, IPF subjects, control subjects, and subjects with other diagnoses not included in the previous disease comparisons. We computed Spearman correlation of Group GSVA score with FEV_1_, % predicted and DLCO, % predicted (Figs. 5 and 6). The Group 1 and Group 3 scores were negative correlated with FEV_1_ and DLCO and the Group 4 score was positively correlated with FEV_1_ and DLCO. The Group 2 score was positively correlated with FEV_1_ but was not correlated with DLCO. There was qualitative visual clustering of both IPF and COPD cases by GSVA score in each group, though with more obvious clustering for IPF subjects than COPD subjects.

### Gene set variation analysis validation in independent COPD and IPF cohorts

We sought to validate our findings in independent COPD and IPF cohorts with available gene expression data. Given the lack of widely available, large scale lung tissue gene expression data and for potential utility as disease biomarkers, we used RNA sequencing and microarray data available from whole blood. There were 1139 COPD cases (GOLD 1–4) and 1459 control subjects from COPDGene for whom RNA-Seq data was available (Additional file 2: Table S7). There were 49 subjects categorized as preserved ratio with impaired spirometry (PRISm) who were also included in the analysis. Cases were older, more likely to be male, more likely to be non-Hispanic white, had lower BMI, less likely to be current smokers, and had higher pack-years. Cases also had lower FEV_1_, % predicted, FEV_1_/FVC ratio, and DLCO, % predicted. There were 57 IPF subjects and 20 control subjects from the Molyneaux et al. study for whom gene expression microarray data was available (Additional file 2: Table S8). There was no difference in age and sex distribution between cases and controls. FVC (% predicted) and DLCO (% predicted) were not reported for control subjects.

GSVA scores were calculated for all subjects in COPDGene and the IPF study using the gene sets derived in LTRC in each of the overlap groups, Groups 1–4. Only the Group 3 GSVA score was significantly different between both COPD cases and controls in COPDGene and IPF cases and controls in the Molyneaux et al. IPF study (Additional file 1: Fig. S3). The Group 2 GSVA score was also significantly different (p < 0.001) between COPD cases and controls in COPDGene, while the Group 1 and Group 4 GSVA scores were significantly different (p < 0.001 and p < 0.01, respectively) between IPF cases and controls in the Molyneaux et al. IPF study.

We tested the association of GSVA scores derived from Group 1–4 genes and clinical traits in COPDGene and the IPF study. Similar to case–control status, the Group 3 GSVA score was significantly associated with worse lung function in both COPDGene (FEV_1_% predicted) and the IPF dataset (FVC % predicted). The Group 2 score was also associated with FEV_1_% predicted as well as the square root of wall area of a hypothetical airway with 10 mm internal perimeter (Pi10) from chest CT scan analysis (Additional file 2: Table S9). The Group 2 score was also nominally associated with DLCO % predicted and mortality in COPDGene, though the p-value did not reach the Bonferroni-corrected significance threshold. The Group 3 score was also only nominally associated with Pi10, percent emphysema, and DLCO % predicted.

In the IPF study, the Group 2 score was also correlated with FVC % predicted (Additional file 1: Fig. S4), but no Group scores were significantly correlated with DLCO % predicted (Additional file 1: Fig. S5).

## Discussion

In this study, we report results from a large sample size of lung tissue RNA sequencing from subjects with COPD and IPF. The overlap patterns of gene expression in lung tissue between the two diseases revealed four distinct groups. We demonstrated that one group of divergent genes in particular, the gene set with increased expression in COPD but decreased expression in IPF (Group 3), was enriched for multiple genes identified by GWAS in IPF and COPD, enriched for important pathways including TNFA signaling via NFKB and TGFB signaling, and overrepresented by myeloid cell genes. These results characterize a unique gene expression signature that may represent specific inflammatory pathways that play a divergent role in the pathogenesis of COPD and IPF. Furthermore, overexpression of this gene signature in blood was associated with reduced lung function in both diseases.

The divergent role of inflammation in COPD and IPF has been independently established but has not been demonstrated using the contrast and overlap approach we employed above. Broadly, immune suppression with systemic corticosteroids has been shown to improve symptoms and reduce length of stay in acute COPD exacerbations [25], but the chronic use of inhaled corticosteroids remains somewhat controversial. On the other hand, immune suppression with corticosteroids and azathioprine was associated with increased mortality in IPF [26].

TNFA signaling represents one such pathway we found to be divergent between COPD and IPF that has been examined in-depth in both diseases. TNFA has a long history in COPD but has ultimately been disappointing as an avenue for pharmacologic intervention. It is thought to play a role in the pathogenesis of COPD via expression from dysregulated monocytes [27] and subsequent apoptosis of cells in the alveolar wall [28], leading to the loss of lung parenchyma and development of emphysema [29]. However, while there may have been a trend toward benefit in a subgroup of subjects with cachexia, a randomized clinical trial of TNFA blockade in subjects with moderate to severe COPD failed to demonstrate a benefit [30]. The role of TNFA in IPF is less clear. While several studies have suggested the role of endogenous TNFA in potentiating the pathogenesis of IPF [31], there may be a protective effect of exogenous or overexpression of TNFA in IPF [32].

TGFB signaling represents another immune-related pathway that has been shown to be associated with both COPD and IPF. TGFB has been studied extensively in IPF and has been linked to inflammatory cell and fibroblast recruitment in the pathogenesis of fibrosis [33]. We found ubiquitous negative enrichment of TGFB signaling in IPF, which may reflect the expression consequences of disease rather than the elements involved in the pathogenesis. On the other hand, there were two distinct gene clusters of TGFB signaling enrichment in COPD, one with positive enrichment and one with negative enrichment. This may signify the presence of TGFB-determined subgroups in COPD [34], defined either by TGFB signaling enrichment or temporal differences of TGFB signaling (i.e. early vs. late). Interestingly, *TGFB2* gene expression, which was one of the genes associated with COPD by GWAS, was decreased in both COPD and IPF and did not belong to the divergent gene signature.

The overrepresentation of myeloid cell genes in the divergent gene expression signature between COPD and IPF further characterizes the opposing role of inflammation in the two diseases. While a small but distinct population of myeloid cells has been associated with IPF [35], several populations of myeloid cells, including alveolar macrophages, dendritic cells, neutrophils, and other cells that make up the foundational population of innate immunity, have been implicated in the chronic inflammation that has been a demonstrated hallmark of COPD [36].

We demonstrated the association of the divergent gene expression signature between the two diseases with worse lung function in LTRC and validated these findings in independent COPD and IPF blood expression data sets. As discussed above, the divergent gene expression signature may represent specific inflammatory pathways that play a divergent role in the branching pathogenesis of the two diseases. Inflammation, both systemic [37, 38] and lung-specific [39], has been associated with worse lung function and progressive decline in COPD. We observed this effect in the COPDGene study replication, with the divergent gene expression signature associated with decreased FEV_1_ and nominally associated with airway wall thickness and mortality. In contrast, while there may be inflammatory aspects of IPF pathogenesis, the data on the role of inflammation in IPF progression are mixed. On one hand, increased innate and adaptive immune infiltrates distinguished rapid progressors from slow progressors in IPF [40]. Similarly, downregulation of CD28 on CD4 T cells has been shown to be associated with a dysregulated, increased immune response associated with progression of IPF [41]. Several genes that are a part of a 52-gene expression score predictive of survival in IPF belong to the costimulatory T cell activation signal [42, 43]. On the other hand, nintedanib [44, 45] and pirfenidone [46], the only pharmacologic interventions that have been shown to slow disease progression in IPF, are thought to affect fibroblast proliferation without a direct effect on inflammation or immune function. Thus, while our findings highlight the divergent gene expression signature with increased expression COPD and decreased expression in IPF, the association of this signature with worse lung function may belie an inflammatory subtype common to both diseases that results in reduced lung function.

While we were able to demonstrate validation of our findings across several analytic modalities and across two independent cohorts, we recognize that there are several limitations to our current study. First, we identified the gene signatures in lung tissue and used gene expression data from blood for validation. Ideally, we would use gene expression from independent lung tissue cohorts for replication. However, gene expression data from blood is much more widely available and in larger sample sizes by several orders of magnitude. In addition, our group has previously shown overlapping disease-specific gene expression across multiple tissue types [47], and peripheral blood is more accessible as a disease biomarker. Second, our use of bulk lung tissue for RNA sequencing has limited cellular precision compared to single cell RNA sequencing. We attempted to address this limitation by including results from a single cell RNA-Seq study in IPF to determine the cell composition of the gene expression overlap groups and cell deconvolution proportions between COPD, IPF, and controls. In addition, given the pathologic heterogeneity associated with both COPD and IPF, we acknowledge that some of the findings may be attributable to differences in lung tissue sampling. However, we attempted to mitigate any differences associated with sampling by using strict, clinical and pathologic composite definitions. Third, subjects in LTRC underwent thoracic surgery for clinical indications, including lung cancer. While the study protocol included clear pathologic margins, any residual field effect could impact gene expression results [48]. Finally, given the advanced stage of disease in the IPF and COPD cases in LTRC, it is difficult to distinguish whether the gene expression in the lung tissue is a reflection of casual pathways or reactive changes. Future studies with early-stage samples may help clarify these results.

## Conclusions

Our study reveals a divergent gene signature with increased expression in COPD and decreased expression in IPF that highlights the opposing role of several inflammatory and immune-related pathways in lung tissue from COPD and IPF. We show that this gene signature is associated with reduced lung function in both COPD and IPF, suggesting the presence of a common inflammatory subtype with increased disease severity.

## Supplementary Information

**Additional file 1.** Supplementary methods and results.

**Additional file 2.** **Supplementary tables. Table S1.** IPF vs. control differential expression results. **Table S2.** COPD vs. control differential expression results. **Table S3.** Overlap genes from COPD and IPF vs. control differential expression. **Table S4.** COPD GWAS loci genes within ± 200 kb in overlapping differential expression genes. **Table S5.** IPF GWAS loci genes within ± 200 kb in overlapping differential expression genes. **Table S6.** Hallmark pathway enrichment. **Table S7.** COPDGene phase 2 RNASeq subject characteristics. **Table S8.** IPF study (Molyneaux et al.) subject characteristics. **Table S9.** GSVA score associations with outcomes in COPDGene.

## Data Availability

Data are available on the NCBI database of Genotypes and Phenotypes (dbGaP), accessions phs000179 and phs000765 (COPDGene) and phs001662 (LTRC).

## Abbreviations

COPD: Chronic obstructive pulmonary disease
IPF: Idiopathic pulmonary fibrosis
GWAS: Genome-wide association study
eQTL: Expression quantitative trait locus
LTRC: Lung Tissue Research Consortium
FEV_1_: Forced expiratory volume in 1 s
FVC: Forced vital capacity
GOLD: Global Initiative for Chronic Obstructive Lung Disease
BMI: Body mass index
DLCO: Diffusion capacity for carbon monoxide
FDR: False discovery rate
GSVA: Gene set variation analysis
TGFB: Transforming growth factor beta signaling
TNFA: Tumor necrosis factor alpha signaling
