# Supplementary material for: Lung tissue shows divergent gene expression between chronic obstructive pulmonary disease and idiopathic pulmonary fibrosis
Source: Respir Res. 2022 Apr 21;23:97. doi: 10.1186/s12931-022-02013-w (PMC9026726; doi:10.1186/s12931-022-02013-w)
Supplement: Supplementary file 1 — Additional file 1. Supplementary methods and results. [file 12931_2022_2013_MOESM1_ESM.docx]

Additional file 1 for:

**Lung tissue RNA sequencing reveals divergent gene expression signaling and inflammatory pathways between IPF and COPD**

AJ Ghosh, BD Hobbs, JH Yun, A Saferali, M Moll, Z Xu, RP Chase, JD Morrow, J Ziniti, F Sciurba, L Barwick, AH Limper, K Flaherty, G Criner, KK Brown, R Wise, FJ Martinez, D McGoldrick, MH Cho, DL Demeo, EK Silverman, PJ Castaldi, NHLBI Trans-Omics for Precision Medicine Consortium, CP Hersh

**Additional Methods**

**Study participants**

One sample per subject was included at random for subjects with duplicate samples where the diagnosis was consistent across samples. Subjects with duplicate samples with discordant diagnoses were excluded. All samples collected as part of the Lung Tissue Research Consortium (LTRC) were collected using a standardized protocol that has been described in the original study design. All samples from the LTRC were sequenced at the Northwest Genomics Center, during Phase 4 of the TOPMed program.

**RNA extraction, Quality control, and Alignment**

An overview of the study design is shown in Figure S1. mRNA sequencing was performed through the NHLBI TOPMed program at the University of Washington. RNA quantification was performed using the Quant-iT RNA assay (Invitrogen) and RNA integrity analysis was performed using a fragment analyzer (Advanced Analytical). Samples were failed for low RNA total amount, concentration, or integrity. Poly-A selection and cDNA synthesis was performed using TruSeq Stranded mRNA kit (Illumina). Final RNASeq libraries were quantified using the Quant-it dsDNA High Sensitivity assay. Our processing pipeline consisted of the following elements: (1) base calls generated in real-time on the NovaSeq6000 instrument (RTA 3.1.5); (2) demultiplexed, unaligned BAM files produced by Picard ExtractIlluminaBarcodes and IlluminaBasecallsToSam were converted to FASTQ format using SamTools bam2fq (v1.4); (3) sequence read and base quality were checked using the FASTX-toolkit (v0.0.13); (4) sequences are aligned to GRCh38 with reference transcriptome GENCODE release 29 using STAR (v2.6.1d). Gene-level expression quantification was generated with RNA-SeQC (v2.3.3) and RSEM (v1.3.1). Sex consistency was confirmed by comparing the expression of Xist and non-pseudoautosomal regions of the Y chromosome samtools with the "view -c" argument and giving genomic ranges corresponding to Xist and subregions of the Y-chromosome.

**Differential Expression Analysis**

Prior to analysis, genes with fewer than 1 count per million reads in more than 50% of subjects were excluded. Differential gene expression was performed using the R/Bioconductor packages limma and voom(E1, E2), where the associations between gene expression levels and IPF vs. control and COPD vs. control were tested. We included current smoking status, as opposed to ever smoking status, as a covariate given the large reported effects of current smoking on lung tissue gene expression and the similarities in gene expression between former and never smokers(E3, E4). Surrogate variables were computed for each phenotype separately, using the sva package(E5).

**Gene Set Enrichment and Pathway Analysis**

We probed the overlap group gene sets for genetic loci, using both the nearest gene and all genes within +/- 200kb, that have been previously identified in GWAS for COPD(E6, E7) and IPF(E8).

Functional enrichment of the overlapping differential gene expression sets was tested using multi-contrast gene set enrichment analysis (mitch), which uses a rank-multiple analysis of variance (MANOVA) approach to identify sets of genes that exhibit joint enrichment across multiple contrasts(E9). We included only genes that carried a HUGO Gene Nomenclature Committee (HGNC) symbol. We chose the Hallmark gene sets and pathways curated by the Molecular Signatures Database (MSigDB) as the reference for annotated gene sets(E10, E11).

**Cell Category Composition**

In the study by Adams et al, each cell type within the cell categories was defined by the top five genes ranked by FDR between average single-cell RNA sequencing (scRNASeq) expression per subject for each cell type vs. the average scRNASeq expression per subject of the other cell types(E12). We arranged genes in each of our overlap gene expression sets by corresponding scRNASeq-derived cell category.

**Cell Type Deconvolution**

We performed cell type deconvolution to determine differences in selected cell types in lung tissue using Bisque. Bisque is a robust cell type proportion estimation method that applies non-negative least-squares (NNLS) regression with an additional sum-to-one constraint on transformed bulk gene expression data, with a paired or unpaired single-cell dataset as a reference(E13). Reference expression profiles for cell types were determined from publicly available lung single-cell RNA sequencing data(E14).

**Additional Results**

**Differential Gene Expression**

As a sensitivity analysis, we reran our differential expression analyses using DESeq2(E15). We used the same model design as in the limma+voom models, adjusting the models for age, gender, race, current smoking status, smoking pack-years, batch, and surrogate variables. There were more differentially expressed genes between both COPD vs. control (5826 genes) and IPF vs. control (12927 genes), after accounting for multiple testing with FDR < 1%. There were 8979 genes (78.4% of the limma+voom results) that overlapped between the limma+voom results and DESeq2 results for IPF and 3792 genes (75.7% of the limma+voom results) that overlapped between the limma+voom results and DESeq2 results for COPD.

**References**

E1. Ritchie ME, Phipson B, Wu D, Hu Y, Law CW, Shi W, Smyth GK. Limma powers differential expression analyses for RNA-sequencing and microarray studies. *Nucleic Acids Res* 2015;43:e47.

E2. Law CW, Chen Y, Shi W, Smyth GK. Voom: Precision weights unlock linear model analysis tools for RNA-seq read counts. *Genome Biol* 2014;15:1–17.

E3. Obeidat M, Dvorkin-Gheva A, Li X, Bossé Y, Brandsma C-A, Nickle DC, Hansbro PM, Faner R, Agusti A, Paré PD, Stampfli MR, Sin DD. The Overlap of Lung Tissue Transcriptome of Smoke Exposed Mice with Human Smoking and COPD. *Sci Rep* 2018;8:11881.

E4. Beane J, Sebastiani P, Liu G, Brody JS, Lenburg ME, Spira A. Reversible and permanent effects of tobacco smoke exposure on airway epithelial gene expression. *Genome Biol* 2007;8:R201.

E5. Leek JT, Johnson WE, Parker HS, Jaffe AE, Storey JD. The SVA package for removing batch effects and other unwanted variation in high-throughput experiments. *Bioinformatics* 2012;28:882–883.

E6. Sakornsakolpat P, Prokopenko D, Lamontagne M, Reeve NF, Guyatt AL, Jackson VE, Shrine N, Qiao D, Bartz TM, Kim DK, Lee MK, Latourelle JC, Li X, Morrow JD, Obeidat M, Wyss AB, Bakke P, Barr RG, Beaty TH, Belinsky SA, Brusselle GG, Crapo JD, de Jong K, DeMeo DL, Fingerlin TE, Gharib SA, Gulsvik A, Hall IP, Hokanson JE, *et al.* Genetic landscape of chronic obstructive pulmonary disease identifies heterogeneous cell-type and phenotype associations. *Nat Genet* 2019;51:494–505.

E7. Hobbs BD, de Jong K, Lamontagne M, Bossé Y, Shrine N, Artigas MS, Wain L V, Hall IP, Jackson VE, Wyss AB, London SJ, North KE, Franceschini N, Strachan DP, Beaty TH, Hokanson JE, Crapo JD, Castaldi PJ, Chase RP, Bartz TM, Heckbert SR, Psaty BM, Gharib SA, Zanen P, Lammers JW, Oudkerk M, Groen HJ, Locantore N, Tal-Singer R, *et al.* Genetic loci associated with chronic obstructive pulmonary disease overlap with loci for lung function and pulmonary fibrosis. *Nat Genet* 2017;49:426–432.

E8. Allen RJ, Guillen-Guio B, Oldham JM, Ma SF, Dressen A, Paynton ML, Kraven LM, Obeidat M, Li X, Ng M, Braybrooke R, Molina-Molina M, Hobbs BD, Putman RK, Sakornsakolpat P, Booth HL, Fahy WA, Hart SP, Hill MR, Hirani N, Hubbard RB, McAnulty RJ, Millar AB, Navaratnam V, Oballa E, Parfrey H, Saini G, Whyte MKB, Zhang Y, *et al.* Genome-wide association study of susceptibility to idiopathic pulmonary fibrosis. *Am J Respir Crit Care Med* 2020;201:564–574.

E9. Kaspi A, Ziemann M. mitch: multi-contrast pathway enrichment for multi-omics and single-cell profiling data. *BMC Genomics* 2020;21:447.

E10. Subramanian A, Tamayo P, Mootha VK, Mukherjee S, Ebert BL, Gillette MA, Paulovich A, Pomeroy SL, Golub TR, Lander ES, Mesirov JP. Gene set enrichment analysis: A knowledge-based approach for interpreting genome-wide expression profiles. *Proc Natl Acad Sci* 2005;102:15545–15550.

E11. Liberzon A, Subramanian A, Pinchback R, Thorvaldsdottir H, Tamayo P, Mesirov JP. Molecular signatures database (MSigDB) 3.0. *Bioinformatics* 2011;27:1739–1740.

E12. Adams TS, Schupp JC, Poli S, Ayaub EA, Neumark N, Ahangari F, Chu SG, Raby BA, DeIuliis G, Januszyk M, Duan Q, Arnett HA, Siddiqui A, Washko GR, Homer R, Yan X, Rosas IO, Kaminski N. Single-cell RNA-seq reveals ectopic and aberrant lung-resident cell populations in idiopathic pulmonary fibrosis. *Sci Adv* 2020;6:eaba1983.

E13. Jew B, Alvarez M, Rahmani E, Miao Z, Ko A, Garske KM, Sul JH, Pietiläinen KH, Pajukanta P, Halperin E. Accurate estimation of cell composition in bulk expression through robust integration of single-cell information. *Nat Commun* 2020;11:1971.

E14. Travaglini KJ, Nabhan AN, Penland L, Sinha R, Gillich A, Sit R V., Chang S, Conley SD, Mori Y, Seita J, Berry GJ, Shrager JB, Metzger RJ, Kuo CS, Neff N, Weissman IL, Quake SR, Krasnow MA. A molecular cell atlas of the human lung from single-cell RNA sequencing. *Nature* 2020;587:619–625.

E15. Love MI, Huber W, Anders S. Moderated estimation of fold change and dispersion for RNA-seq data with DESeq2. *Genome Biol* 2014;15:550.

**Figure S1: Overview of Study Design**

**
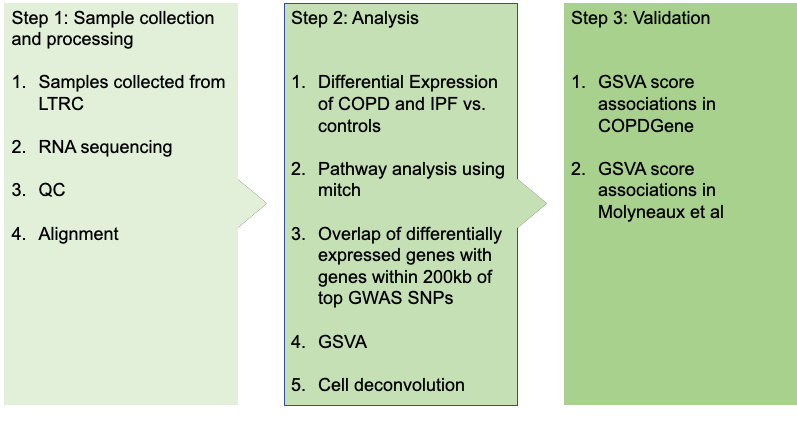
**

**Figure S2: Principal component analysis (PCA) plots for lung tissue RNA sequencing data**


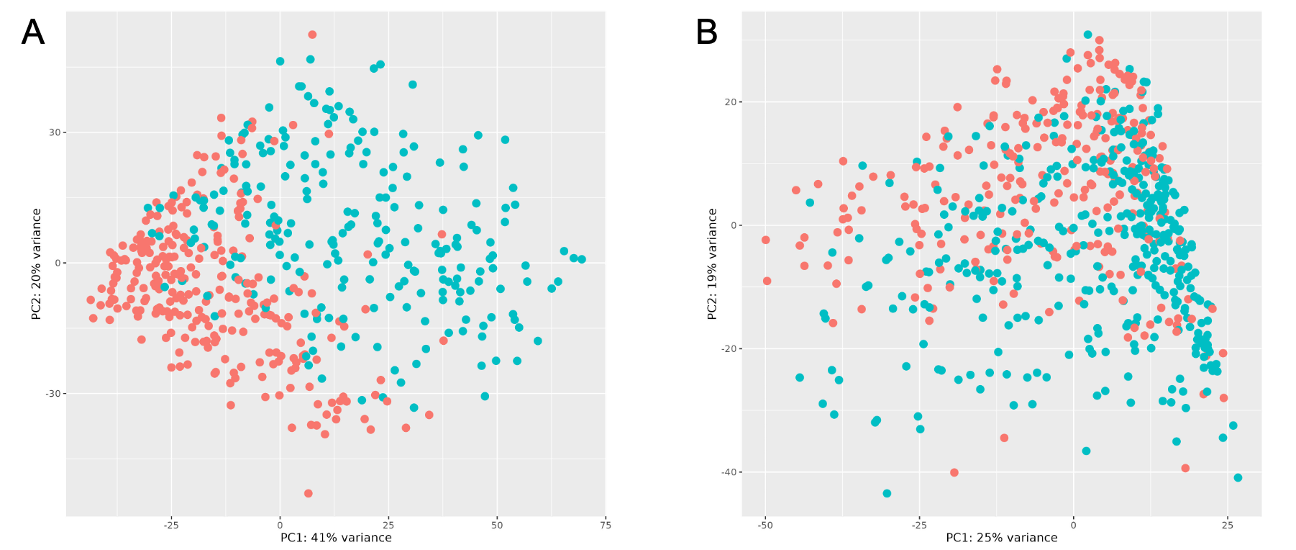


Principal component analysis (PCA) plots for lung tissue RNA sequencing data. **A** PCA plot for control samples (orange) vs. IPF samples (blue). **B** PCA plot for control samples (orange) vs. COPD samples (blue).

**Figure S3: Volcano plots of differential expression results**

**
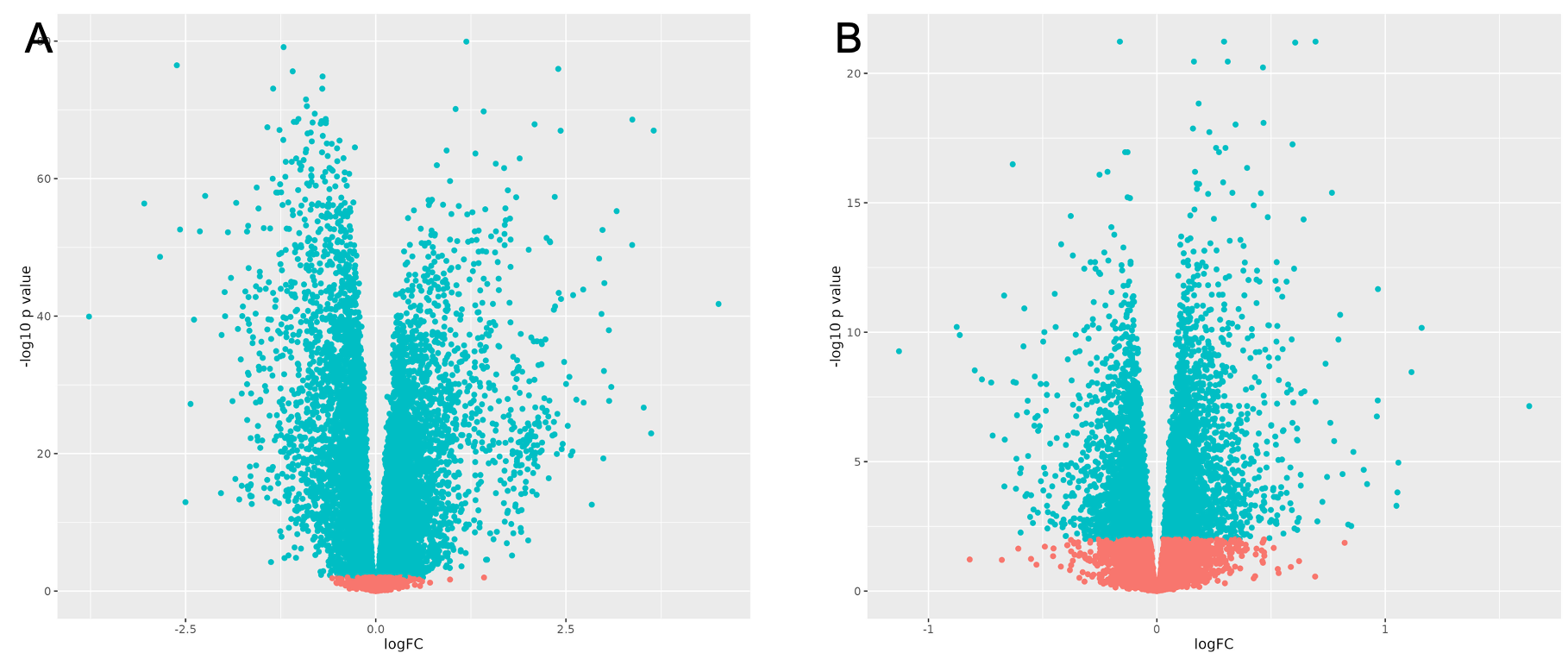
**

Volcano plots of differential expression results. FDR < 0.01 results shown in blue and results that did not meet FDR threshold are shown in orange. **A** Volcano plot of IPF vs control differential expression results. **B** Volcano plot of COPD vs control differential expression results.

**Figure S4: Log-transformed expression of select differentially expressed genes**

**
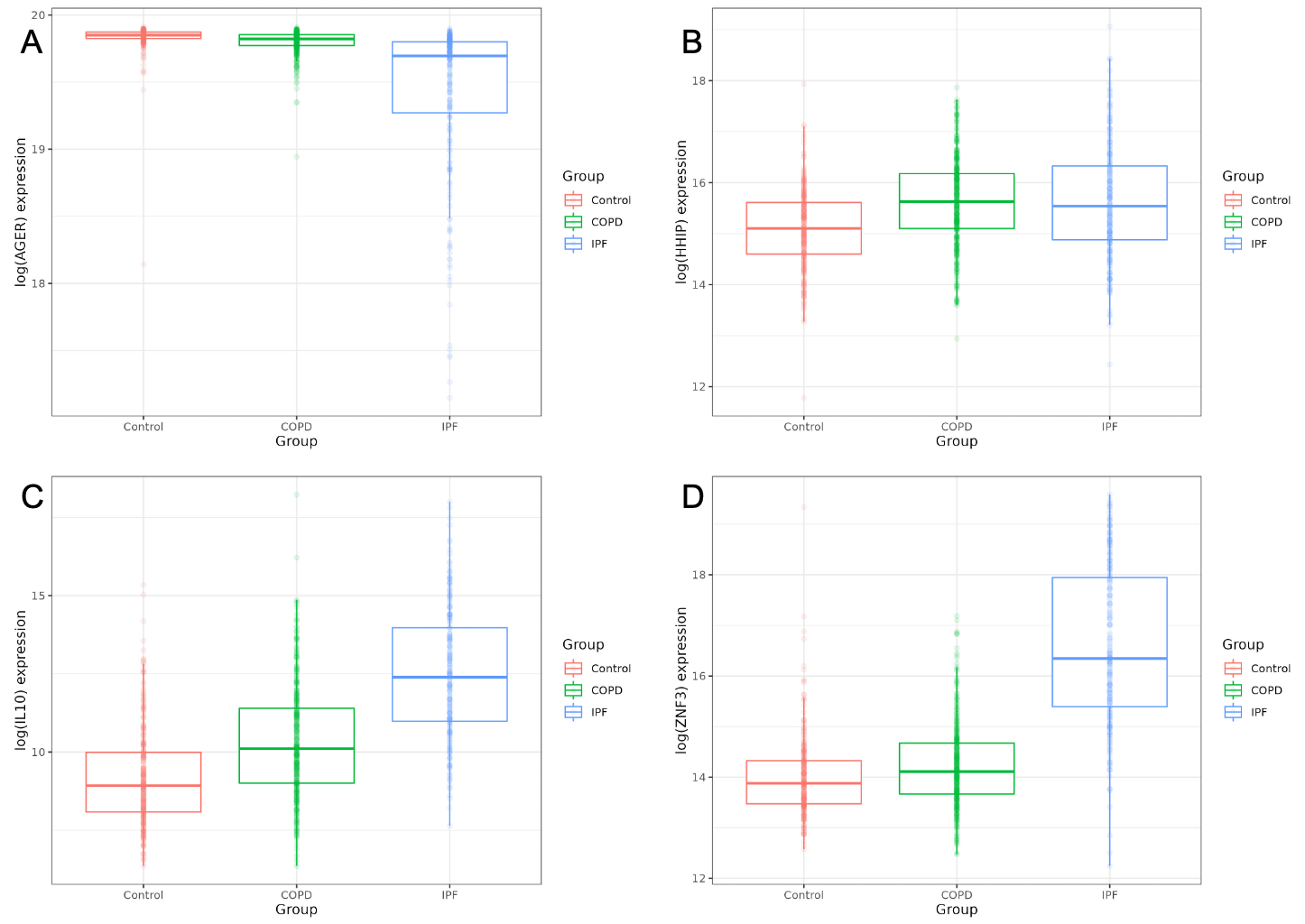
**

Boxplots of log-transformed expression data of select differentially expressed genes across COPD, IPF, and control samples. **A** Log-transformed expression of *AGER*. **B** Log-transformed expression of *HHIP*. **C** Log-transformed expression of *IL10*. **D** Log-transformed expression of *ZNF3*.

**Figure S5: Association of GSVA scores from overlap groups with case-control status in COPDGene and IPF study (Molyneaux et al)**

**
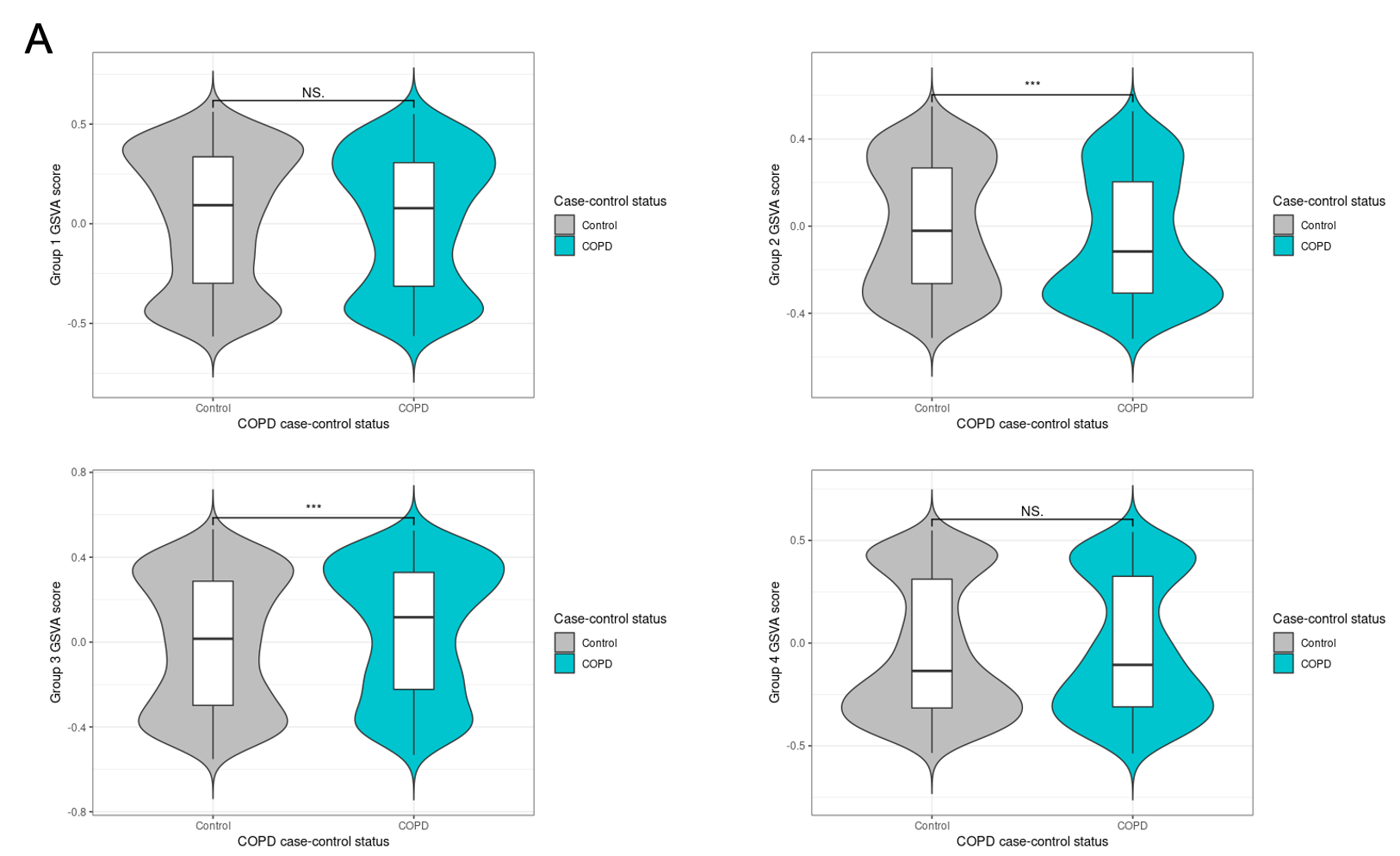
**


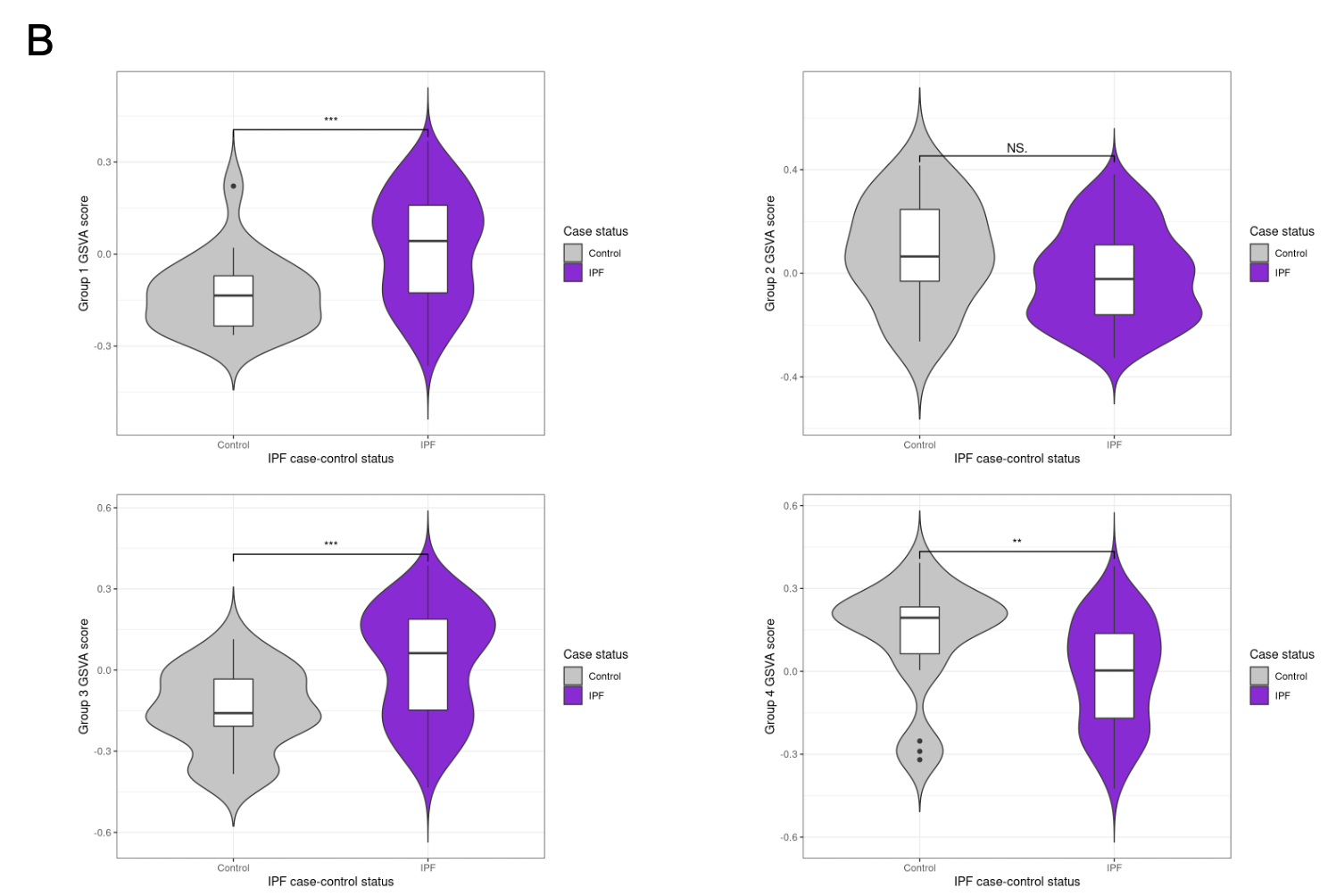


Box-whisker and violin plots of GSVA scores by case-control status shown for GSVA score from each overlap group. Number of * corresponds to significance level: * = p < 0.05, ** = p < 0.01, *** = p < 0.001. Group 1: genes with increased expression in IPF and COPD; Group 2: genes with increased expression in IPF but decreased expression in COPD; Group 3: genes with decreased expression in IPF but increased expression in COPD; Group 4: genes with decreased expression in IPF and COPD. **A** Box-whisker and violin plots by COPD case-control status for GSVA scores from each overlap group in COPDGene. **B** Box-whisker and violin plots by IPF case-control status for GSVA scores from each overlap group in Molyneaux et al. GSVA: gene set variation analysis; COPD: chronic obstructive pulmonary disease; IPF: idiopathic pulmonary fibrosis.

**Figure S6: Association of GSVA scores from overlap groups with FVC % predicted in IPF study (Molyneaux et al)
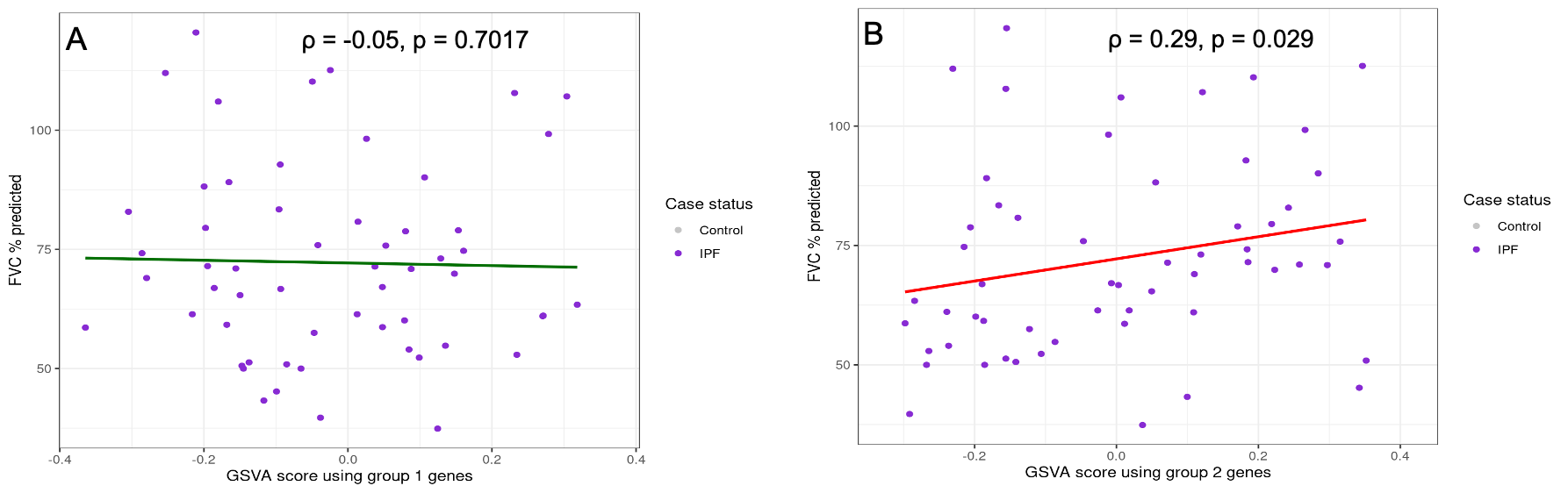
**

**
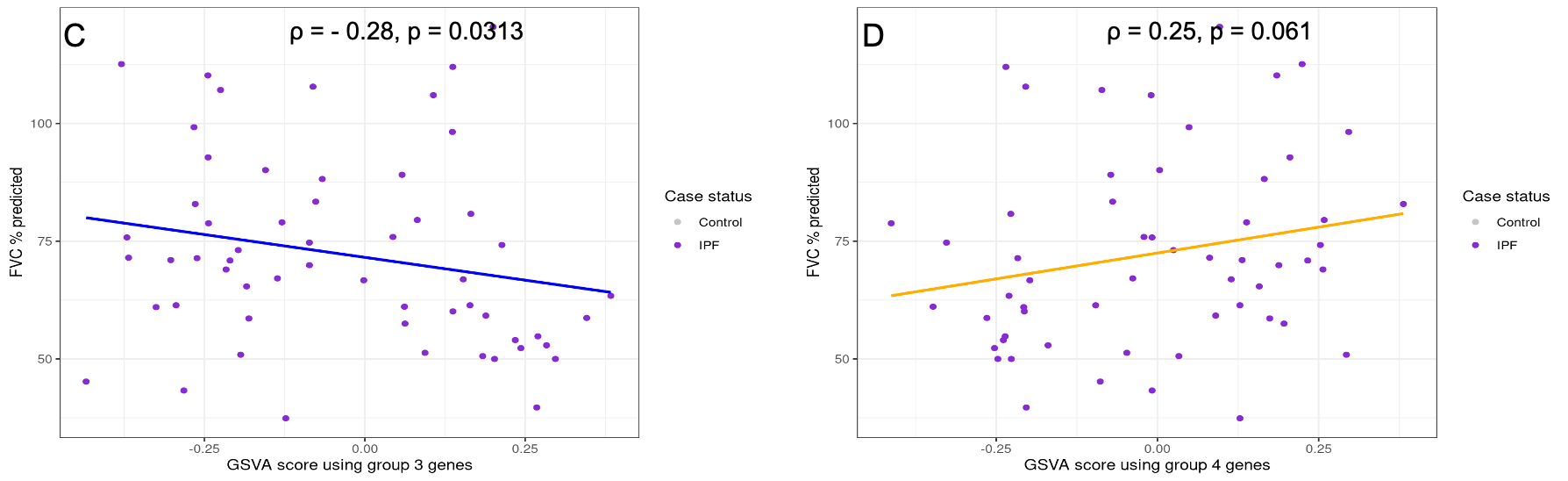
**

Scatterplots with trend lines of association of GSVA scores from overlap groups with FVC % predicted in Molyneaux et al subjects. FVC % predicted data not reported for control subjects. Trend lines colored to represent overlap group. Spearman correlation coefficient and p value shown for each association. Group 1: genes with increased expression in IPF and COPD; Group 2: genes with increased expression in IPF but decreased expression in COPD; Group 3: genes with decreased expression in IPF but increased expression in COPD; Group 4: genes with decreased expression in IPF and COPD. **A** Association of GSVA score from Group 1 genes with FVC % predicted. **B** Association of GSVA score from Group 2 genes with FVC % predicted. **C** Association of GSVA score from Group 3 with FVC % predicted. **D** Association of GSVA score from Group 4 with FVC % predicted. GSVA: gene set variation analysis; IPF: Idiopathic Pulmonary Fibrosis; FVC: forced vital capacity.

**Figure S7: Association of GSVA scores from overlap groups with DLCO % predicted in IPF study (Molyneaux et al)**

**
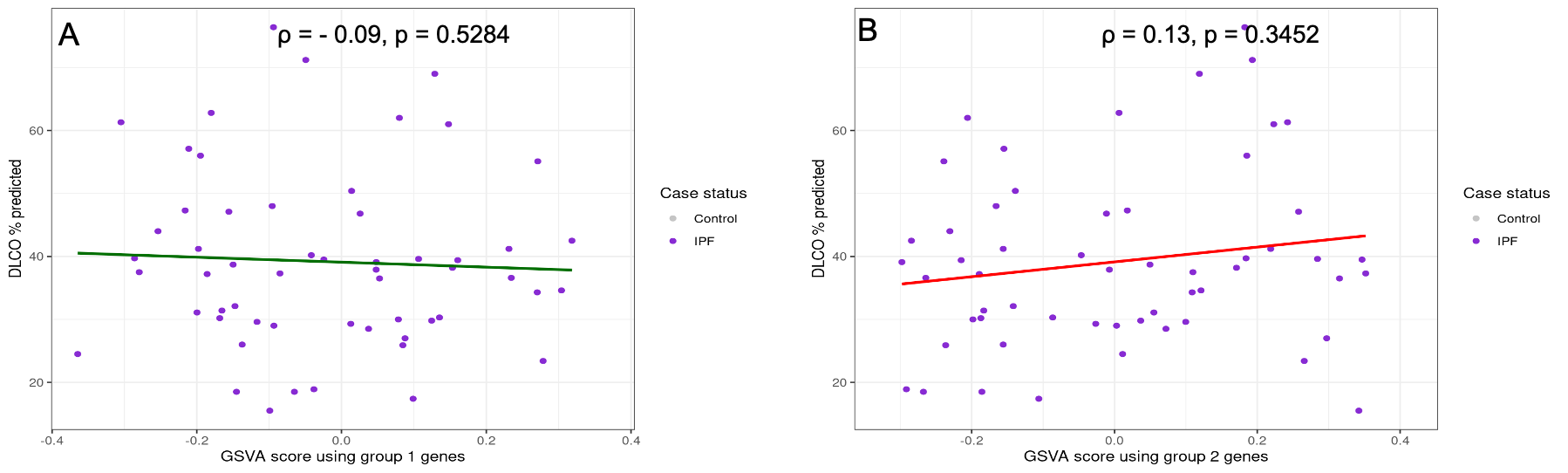
**

**
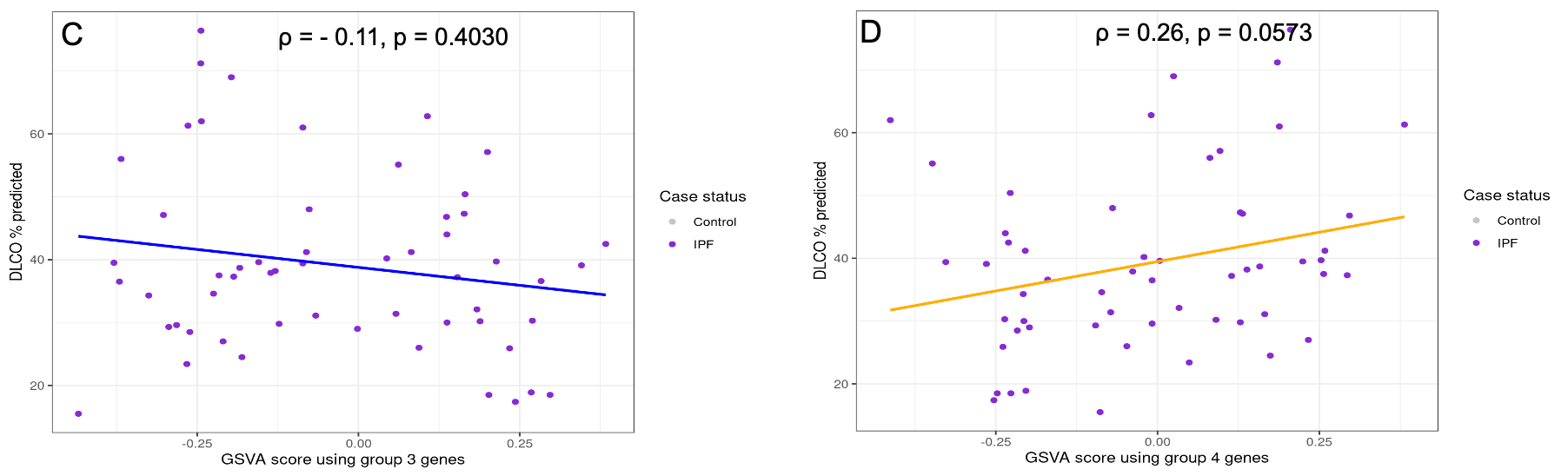
**

Scatterplots with trend lines of association of GSVA scores from overlap groups with DLCO % predicted in Molyneaux et al subjects. DLCO % predicted data not reported for control subjects. Trend lines colored to represent overlap group. Spearman correlation coefficient and p value shown for each association. Group 1: genes with increased expression in IPF and COPD; Group 2: genes with increased expression in IPF but decreased expression in COPD; Group 3: genes with decreased expression in IPF but increased expression in COPD; Group 4: genes with decreased expression in IPF and COPD. **A** Association of GSVA score from Group 1 genes with DLCO % predicted. **B** Association of GSVA score from Group 2 genes with DLCO % predicted. **C** Association of GSVA score from Group 3 with DLCO % predicted. **D** Association of GSVA score from Group 4 with DLCO % predicted. GSVA: gene set variation analysis; IPF: Idiopathic Pulmonary Fibrosis; DLCO: diffusion capacity for carbon monoxide.
